# Prevalence of High Fat Sugar Salt Products, Labeling Characteristics, and Categories of Foods Sold within In-Store Restricted Areas: A Survey in 3 UK Supermarkets after the 2022 Implementation of the Food (Promotion and Placement) Regulations

**DOI:** 10.1016/j.cdnut.2024.104509

**Published:** 2024-11-20

**Authors:** Ella Hurst, Sally G Moore, Lewis W Wallis

**Affiliations:** School of Food Science and Nutrition, University of Leeds, Leeds, West Yorkshire, United Kingdom

**Keywords:** retail food environments, supermarkets, front-of-pack nutrition labeling, policy, product promotions, less healthy, HFSS, nutrient profile

## Abstract

**Background:**

Regulations restricting the promotion of some less-healthy products high in fat, sugar, or salt (HFSS) within “restricted areas” (RAs) of supermarkets came into force in October 2022 in England.

**Objectives:**

To evaluate the prevalence of HFSS products and front-of-pack nutrition labeling (FOPNL) characteristics of foods sold within RAs in a sample of supermarket stores.

**Methods:**

A cross-sectional survey of products in RAs in 3 supermarkets was undertaken from November 2022 to February 2023 using photographs, recording the display of FOPNL. Identified via the online supermarket, product nutrition and ingredient data were collected and used to categorize each as either “in” or out-of-scope of the regulations. The UK Nutrient Profiling Model was used to determine product HFSS status and the FOPNL multiple traffic light criteria used to calculate the number of inherent red traffic lights (iRTLs) possessed. Prevalence of HFSS, FOPNL, and iRTLs were calculated as a proportion (%) of total products. Associations between these characteristics were explored using chi-squared tests.

**Results:**

A total of 86 RAs were identified across the 3 stores, of which 32 displayed 679 food products. Most of these products fell into categories considered out-of-scope of the regulations (64%, *n =* 435) with prevalence of HFSS at 42% (282 of 435 products). For products within in-scope categories, 17% were HFSS (42 of 245). Half of all included products (53%, *n =* 357) displayed FOPNL, and 16% possessed 1–3 iRTLs, including both HFSS and non-HFSS items. HFSS products in categories in-scope of the regulations were less likely to display FOPNL compared with non-HFSS products (X^2^ =25, *P* < 0.001).

**Conclusions:**

After the implementation of The Food (Promotion and Placement) Regulations, foods sold in RAs of 3 supermarkets included those in categories in- and out-of-scope, a variable prevalence of less-healthy (HFSS) products, display of FOPNL, and possession of iRTLs. Findings and approach support future impact evaluation.

## Introduction

Consumption of unhealthy diets contributes to the global burden of diet-related diseases, including obesity [1]. To tackle this problem, attention has turned to the role of food environments where people interact with the wider food system to acquire and consume foods [2]. Within retail food environments (that is, supermarkets), policy initiatives that aim to support consumers in making healthier choices include the display of interpretative front-of-pack nutrition labeling (FOPNL) on products [3]. This information is intended to improve consumers’ food choices by indicating “high” (red) and lower (amber or green) levels of specific nutrients of public health concern, including fat, saturated fat, sugar, and salt [4,5]. However, there are other ways in which retail food environments can influence consumers to buy specific food products, such as by using product promotions. Promotions include offers on product price (that is, temporarily reducing the price), volume (that is, offering a discount for purchasing multiple items), or placement (that is, placing products physically closer to the customer, such as at the end-of-aisles) [[6], [7], [8]]. In the United Kingdom, it is thought that ends-of-aisles contribute heavily to consumers’ overall spend and around 30% of total retail sales [9,10]. Promotions are therefore considered likely to promote overconsumption of certain types of products or nutrients of public health concern, particularly if the food on promotion may be considered “less healthy” [11,12]. With this in mind, the UK Government considers that “retail promotional environments do not always align with government healthy eating guidelines, and as such this makes it harder for people to make healthier choices when shopping” [11,10].

Because of their proximity to the customer, the ends-of-aisles and other key in-store locations such as checkouts, queuing areas, store entrances, etc. are therefore ideal targets for policies that restrict the display of less-healthy products and promote healthier items. Review evidence synthesizing several real-life in-store studies shows a positive impact on sales when stores promote “healthier” products [7]. Such products include those from categories including fruit and vegetables, promoted using a combination of price and placement/proximity (that is, on a specific shelf, front of store, at eye-level), and compared with preintervention or control stores [7]. In contrast, there is less evidence regarding the restriction of promotion of less-healthy food products by location, yet research shows some impact on sales [7]. This includes research in the United Kingdom that reported a decrease in sales of less-healthy common checkout products after implementation of voluntary supermarket policies to restrict “high fat, sugar salt” (HFSS) products at these in-store locations [13]. Overall, the available research supports development of the new Food (Promotion and Placement) Regulations (England) 2021, which now restrict the appearance of specific categories of HFSS products displayed within prominent in-store locations [14]. These “restricted areas” (RAs) include store entrances, aisle ends, and checkouts of stores with areas over 185.8 m^2^ [14].

The 13 product categories that are “in-scope” of these new regulations include major contributors to UK population intakes of nutrients of public health concern (that is, saturates, sugars, salt, etc.). Identified using dietary intake data, in-scope categories include those contributing to sugar intakes, for example, sugar-sweetened soft drinks, breakfast cereals, yogurts, biscuits, cakes, and confectionery [15]. In addition, the regulations define which product types, within these in-scope categories, are considered “less healthy” by using the UK Nutrient Profile Model (NPM) [16], to define HFSS products on the basis of their nutrient and ingredient content [16]. In contrast, the UK multiple traffic light (MTL) FOPNL criteria identifies “high” (red), “medium” (amber), or “low” (green) levels of 4 nutrients (fat, saturates, sugars, salt) that are intended to be displayed on the FOPNL, albeit voluntarily [5].

In the literature, there exists heterogeneity across studies that have evaluated in-store interventions in the types of “healthier” products and categories they used (that is, bananas, “low calorie” foods, popcorn, etc.) [7]. However, 1 study has previously found a reduction in “less-healthy” (HFSS) products at supermarket checkouts in stores with (voluntary) policies on restricting such products, compared with those without [17]. In this case, classification of the products as “HFSS” was based on the evaluation of the content “per 100 g” of energy, saturates, sugar, sodium, protein, fiber, and fruit/vegetables/nuts, as required in the UK NPM. Besides this, there is currently little evidence available that specifically evaluates the HFSS status, nutritional composition, and FOPNL of products now promoted in UK supermarkets, including those stores newly experiencing regulatory restrictions. This study aims to evaluate specific product-level aspects of supermarket stores after the implementation of the new regulations with respect to these and other policies such as FOPNL, also in operation in the United Kingdom. We address 3 specific research questions:

1) What types of products (and categories) are being sold in RAs in-store, and what is the prevalence of less healthy (HFSS) products?
2) What is the prevalence of displayed FOPNL on products sold in these locations?
3) How do the products sold in these locations evaluate nutritionally using other UK policy-relevant nutritional profiling criteria, such as the number of red traffic lights?

## Methods

### Design and setting

**FIGURE 1:**
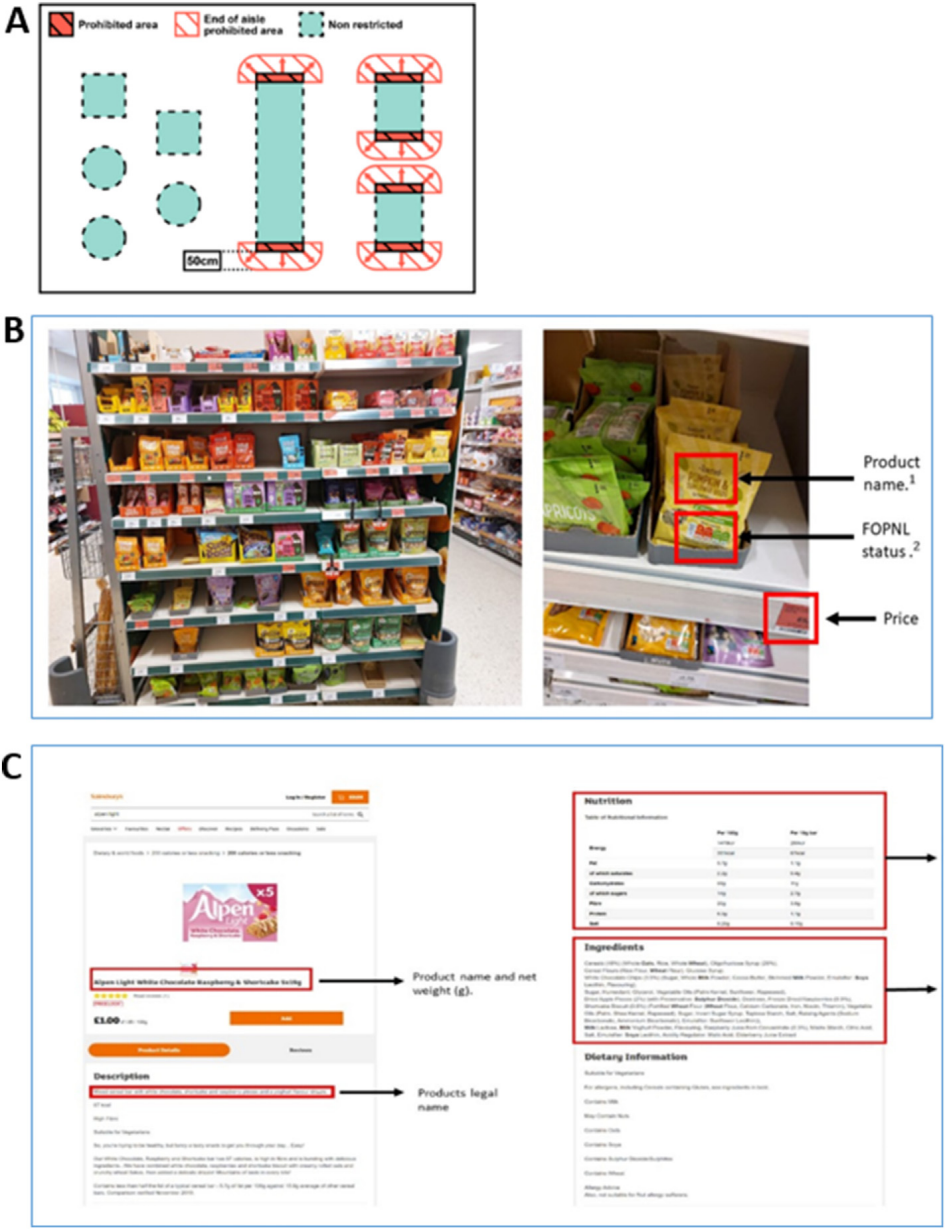
Restricted areas and product-level data collection. (A) Illustration of regulated in-store ends-of-aisles (in red cross hatched) [14]. (B) Photographs of an in-store restricted area (aisle-end, left), with arrows indicating aspects of product data collected [i.e. product name, front-of-pack nutrition label (FOPNL) display]. (C) Supermarket website product information page, with red boxes and arrows indicating specific product-level data collected, including product name, legal name, nutrition information per 100 g, serving size/number of servings, and ingredient declaration.

After implementation of the regulations, a cross-sectional survey of products sold in RAs within 3 in-store supermarket environments was undertaken during November 2022–February 2023. Three supermarket stores from major retailers (Tesco, Sainsbury's, and Morrisons) were selected within the city of Leeds, West Yorkshire (United Kingdom), based on store eligibility given in the regulations [14]. RAs were identified manually by the researcher during store visits, based on the 5 types of RAs for which specific definitions are given in the regulations (for example, aisle ends defined as shelves or items displayed perpendicular to the aisle or ≤50 cm from the aisle-end) (see Supplemental Table 1 and Figure 1A) [14].

### Procedure

To evaluate the nature of products sold in in-store RAs in relation to the regulations, we developed a 7-step approach to collect data on RAs and each product sold within these (Table 1) [14]. Visiting each store, the researcher (EH) first identified and recorded, by taking several photographs of, all potential RAs. Distances were approximated by eye, not measured. Data captured did not include nearby hanging items or baskets/add-ons.

**TABLE 1:** Step-wise approach to evaluating products in-store restricted areas (RAs) in relation to the Food (Promotion and Placement) Regulations 2021 [14].

	Description	Relevant heading number in the regulations
**1**	Select retailer and store	4. Qualifying businesses (i.e. employee count and store size)
**2**	Visit store and manually identify and photograph RAs	7. Location promotions: 5 types of in-scope RAs (illustrated in Annex C Schedule 1)
**3**	Determine from photographs if RAs contain food or non-food (i.e. non-food, alcohol) products	3. What food is in-scope? (i.e. food and drink only)
**4**	For included RAs (i.e. displaying food items), use photographs to identify name, description, and brand of each product and for the purposes of this study, record if FOPNL displayed (Figure 1B)	3. UK Nutrient Profiling Model Technical Guidance referred to
**5**	For each product in included RAs, obtain product-level nutrition (per 100 g) and ingredient information from the corresponding online supermarket information webpages (Figure 1C).	
**6**	Determine if the product is “less healthy” (HFSS) by using regulation’s specified Nutrient Profiling Technical Guidance	
**7**	Determine the product category each product falls into, using those categories that are considered “in-scope” of the regulations	1. Regulation Annex: foods in-scope: 13 categories

Abbreviations: FOPNL, front-of-pack nutrition labeling; HFSS, high fat, sugar, and salt.

### Data collection of product-level information

The photographs showing products in RAs were first enlarged and used by the researcher to record each product’s name, brand, price and on-pack display, and type of FOPNL (that is, MTLs or other scheme) (Figure 1B). This information was used to search for the individual product on the corresponding supermarket website to formally identify the product and collect data on its legal name, ingredients, serving size, and nutritional composition per 100 g (Figure 1C). To evaluate product-level healthfulness using both the UK NPM and UK MTL FOPNL criteria, specific product-level nutrition information was collected including the content of energy (kcal/kJ), total fat (g), saturated fat (g), sugars (g), and salt (g). Where any of the required nutrition information elements (for example, fiber for which declaration is voluntary) were missing from the product webpage, values were recorded and analyzed as “0.” Products (*n =* 3) that did not include any nutrition information on their product webpage were removed from the sample because onward analysis with nutritional evaluation could not be performed. Finally, to assign each product to a specific category, either in or out of scope of the 13 categories given in the regulations, the product’s legal name and ingredient information was used by the researcher, together with the definitions and examples of what was considered in or out of scope according to published industry and government guidance [14,18] (for details see Supplemental Tables 2 and 3). A screenshot of each product’s full product webpage was taken using the Google Chrome extension GoFullPage and product-level data from this was manually inputted into the Microsoft Excel (V16.52) spreadsheet used for analysis.

### Data analysis

#### Application of UK NPM and MTL FOPNL nutrient criteria to product-level data

We used 2 current UK measures of product-level healthfulness to evaluate each product’s nutritional composition in the RAs. These were the UK NPM [16] and UK MTL FOPNL criteria [5]. Briefly, the UK MTLs evaluate each product’s content of fat, saturates, sugars, and salt as green/amber/red on a “per 100 g” and “per serving” basis [5], whereas the UK NPM computes a score based on the “per 100 g” delivery of energy, saturates, sugars, sodium, fiber, protein, fruit/vegetables, and nuts [16]. The UK NPM score is used to classify products as non-HFSS (that is, <4) or HFSS in line with the regulations. In this study, the UK MTL criteria were used to evaluate each product’s possession of 0–4 inherent red traffic lights (iRTLs) (inherent, regardless of their voluntary display on-pack). First, each product’s UK NPM score was calculated using Excel formulas [16] wherein calculations used data collected on each product’s nutritional composition per 100 g (that is, energy, protein, fiber, total sugars, saturated fat, sodium), as well as declared or estimated percentages of fruit, vegetables, and nuts from the declared ingredient listing. For ingredient declarations (*n =* 15) where no quantitative percentage (%) information was given for fruit, vegetable, and nut ingredients, values were estimated according to other ingredient quantities and order of appearance in the ingredient listing, using researcher judgment. In keeping with the classifications used in the UK NPM, foods were classified as less healthy (that is, HFSS) if they scored 4 or more, and drinks if they scored 1 or more [16]. Then, to calculate each product’s possession of iRTLs for fat, saturated fat, sugars, and salt, the UK MTL color coding criteria [5] was used, together with product-level information on total fat, saturated fat, total sugar, and salt content “per 100 g” and “per serving,” and traffic light colors were assigned using an Excel formulas based on the UK MTL guidance [5]. For each product, the number of “red” traffic lights inherently possessed by each product (iRTL) was then calculated.

#### Prevalence of HFSS products, FOPNL, and iRTL across included RAs

RAs included in the analysis were those identified and displayed food products only. Identified RAs displaying non-foods (that is, flowers), ingredients (that is, yeast), alcohol, or no products were also photographed but could not be included in the onward product-level analysis. Prevalence of HFSS, FOPNL, and 1–3 iRTLs was calculated as percentage of the included products of the total sample (or of the specified group, store, or category). All percentages were rounded to the nearest whole number. Chi-squared tests were used to explore associations between the proportion of HFSS compared with non-HFSS products and the prevalence of FOPNL, or the proportion of products with 0, 1, 2, or 3 iRTLs. Degrees of freedom (df) and *P* values are reported, with the latter considered significant when <0.05.

## Results

### In-store RAs: identification and characterization

Across the 3 stores, a total of 86 RAs were identified and photographed, of which 54 were excluded from the analysis because they displayed non-food or ingredient products, the nature of which was not in-scope of the regulations and unable to be evaluated with the UK NPM (Table 2) [5,14,16]. These excluded RAs displayed signage/boards only without products (*n =* 12), alcohol (*n =* 7), or other non-food or ingredient (flour, yeast, etc.), and household products (*n =* 35). Of the 32 RAs included in the analysis, the majority were “end-of-aisle” types (91%, *n =* 29), with some in “queuing areas” (*n =* 2) or at “till points” (*n =* 1). The number of RAs included varied across the 3 sampled stores, with 75% (*n =* 24) from store 1 (Morrisons), 9% (*n =* 3) from store 2 (Sainsbury's), and 16% (*n =* 5) from store 3 (Tesco) (Table 2). Within the included RAs across all 3 stores, a total of 676 products were identified. Most were from categories that were considered out of scope of the regulations (*n =* 435, 64%) (Table 2).

**TABLE 2:** Number of restricted areas identified and included in the study and associated products in-scope of the regulation, by store.

Store	Total number of restricted areas identified	Number of restricted areas included in analysis	Total number of products in included RAs (percentage of all products included in analysis)	Number of products in in-scope categories (% of products in that store)	Those products in out-of-scope categories (% of total for each store)
**Store 1**	58	24	433 (64%)	142 (33%)	291 (67%)
**Store 2**	7	3	130 (19%)	55 (42%)	75 (58%)
**Store 3**	21	5	113 (17%)	44 (39%)	69 (61%)
**Total (% of total sample**^1^**)**	86	32	676 (100%)	245 (36%^a^)	435 (64%^a^)

Abbreviations: RA, restricted area.

1 Percentage of the total sample.

### Products in RAs: category types and prevalence of “less healthy” (HFSS)

**FIGURE 2:**
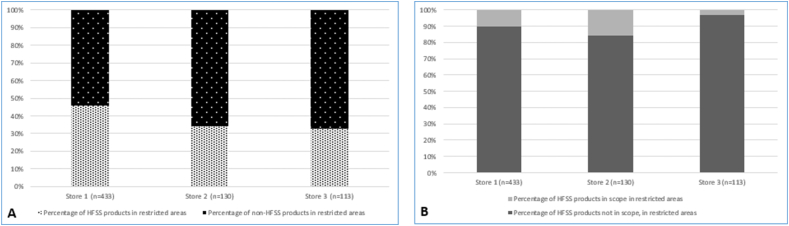
Products in restricted areas characterized by HFSS status and by products within in/out-of-scope categories. (A) Distribution of HFSS and non-HFSS status, by store. (B) Distribution of HFSS products according to categories in or out-of-scope of the regulations, by store. HFSS, high fat, sugar, and salt.

Across included RAs, 245 products spanned all 13 categories that are “in-scope” of the regulations (that is, savory snacks, ready meals) (Table 3). Prevalence of less-healthy (HFSS) items within products in categories in-scope of the regulations was 17% (*n =* 42), with variations between stores (Figure 2) and by category [for example, 41% (*n =* 9) of confectionery products, 39% (*n =* 5) of yogurts, and 0% of breakfast cereals were HFSS] (Table 3). Prevalence of HFSS products across all out-of-scope categories was 55% (*n =* 240) with variations also seen between stores (Figure 2) and by category [for example, 98% (*n =* 45) of cheese and cheese dippers and 100% (*n =* 15) of fat and fat spreads were HFSS] (Table 3). Across included RAs, the prevalence of HFSS products within all in- and out-of-scope categories was 42% (*n =* 282). There was a significantly greater proportion of HFSS (compared with non-HFSS) products in out-of-scope product categories (240 compared with 195 of 435), compared with in-scope product categories (42 compared with 203 of 245) (X^2^ = 93.5, df = 1, *P* < 0.001).

**TABLE 3:** Prevalence of HFSS, FOPNL, and possession of 0, 1, 2, or 3 iRTLs, by product-category.

	Products	HFSS status^1^		FOPNL display on pack^2^			Number of inherent **r**ed traffic lights (iRTLs)^3^			
		HFSS	Non-HFSS^2^	Multiple traffic lights	Other FOPNLs	Total	0	1	2	3
**Product categories in-scope of the regulations**^4^										
**1 Soft drinks**	27	2 (7%)	25 (93%)	8 (30%)	4 (15%)	12 (44%)	15 (56%)	12 (44%)	0 (0%)	0 (0%)
**2 Savory snacks**	58	16 (28%)	42 (72%)	18 (31%)	6 (10.4%)	24 (41%)	55 (95%)	2 (3%)	1 (2%)	0 (0%)
**3 Breakfast cereals**	15	0 (0%)	15 (100%)	15 (100%)	0 (0.0%)	15 (100%)	15 (100%)	0 (0%)	0 (0%)	0 (0%)
**4 Confectionery**	22	9 (41%)	13 (59%)	1 (4.5%)	3 (14%)	4 (18%)	22 (100%)	0 (0%)	0 (0%)	0 (0%)
**5 Ice cream and similar**	3	1 (33%)	2 (67%)	1 (33%)	1 (33%)	2 (67%)	3 (100%)	0 (0%)	0 (0%)	0 (0%)
**6 Cakes and cupcakes**	15	0 (0%)	15 (100%)	2 (13%)	3 (20%)	5 (33%)	15 (100%)	0 (0%)	0 (0%)	0 (0%)
**7 Sweet biscuits or bars**	33	6 (18%)	27 (82%)	0 (0%)	18 (55%)	18 (55%)	33 (100%)	0 (0%)	0 (0.0%)	0 (0%)
**8 Morning goods**	2	0 (0%)	2 (100%)	2 (100%)	0 (0.0%)	2 (100%)	2 (100%)	0 (0%)	0 (0%)	0 (0%)
**9 Desserts and puddings**	6	0 (0%)	6 (100%)	3 (50%)	0 (0.0%)	3 (50%)	6 (100%)	0 (0%)	0 (0%)	0 (0%)
**10 Sweetened yogurt**	13	5 (39%)	8 (61%)	0 (0%)	5 (39%)	5 (39%)	13 (100%)	0 (0%)	0 (0%)	0 (0%)
**11 Pizza**	6	1 (17%)	5 (83%)	6 (100%)	0 (0%)	6 (100%)	5 (83%)	1 (17%)	0 (0%)	0 (0%)
**12 Fried potatoes**	1	0 (0%)	1 (100%)	1 (100%)	0 (0%)	1 (100%)	1 (100%)	0 (0%)	0 (0%)	0 (0%)
**13 Ready to cook meals or meal centers**	44	2 (5%)	42 (96%)	32 (73%)	0 (0%)	32 (73%)	35 (80%)	4 (9%)	1 (2%)	4 (9%)
**Total (for products in in-scope categories)**	245 (100%)	42 (17%)	203 (83%)	89 (36%)	40 (16%)	129 (53%)	220 (90%)	19 (8%)	2 (1%)	4 (2%)
**Product categories out of scope of the regulations**^5^										
**Nuts and seeds**	58	31 (53%)	27 (47%)	21 (36%)	2 (3%)	23 (40%)	43 (74%)	5 (9%)	10 (17%)	0 (0%)
**Processed meat/meat**	49	42 (86%)	7 (14%)	23 (47%)	1 (2.04%)	24 (49%)	40 (82%)	3 (6%)	4 (8%)	2 (4%)
**Cheese and cheese dippers**	45	44 (98%)	1 (2%)	13 (29%)	12 (27%)	25 (56%)	37 (82%)	8 (18%)	0 (0%)	0 (0%)
**Beverages**	42	0 (0%)	42 (100%)	22 (52%)	1 (2%)	23 (55%)	42 (100%)	0 (0%)	0 (0%)	0 (0%)
**Party food**	34	20 (59%)	14 (41%)	34 (100%)	0 (0%)	34 (100%)	33 (97%)	1 (3%)	0 (0%)	0 (0%)
**Instant pasta, noodles, rice**	24	6 (25%)	18 (75%)	9 (38%)	9 (38%)	18 (75%)	9 (38%)	3 (13%)	10 (42%)	2 (8%)
**Fruit**	24	9 (38%)	15 (63%)	5 (21%)	2 (8%)	7 (29%)	24 (100%)	0 (0%)	0 (0%)	0 (0%)
**Plain carbohydrates**	23	0 (0%)	23 (100%)	20 (87%)	0 (0%)	20 (87%)	23 (100%)	0 (0%)	0 (0%)	0 (0%)
**Fat and fat spread**	15	15 (100%)	0 (0%)	2 (13%)	0 (0%)	2 (13%)	15 (100%)	0 (0%)	0 (0%)	0 (0%)
**Vegetables/Pickles**	14	5 (36%)	9 (64%)	1 (7%)	0 (0%)	1 (7%)	12 (86%)	2 (14%)	0 (0%)	0 (0%)
**Dessert ingredients/decoration**	13	12 (92%)	1 (8%)	3 (23%)	0 (0%)	3 (23%)	5 (39%)	8 (62%)	0 (0%)	0 (0%)
**Spreads and sweet spreads**	13	13 (100%)	0 (0%)	3 (23%)	4 (31%)	7 (54%)	10 (77%)	0 (0%)	2 (15%)	1 (8%)
**Gravy and stock**	13	6 (46%)	7 (54%)	6 (46%)	0 (0%)	6 (46%)	13 (100%)	0 (0%)	0 (0%)	0 (0%)
**Pies, pasties and Yorkshire Puddings**	12	10 (83%)	2 (17%)	1 (8%)	0 (0%)	1 (8%)	1 (8%)	0 (0%)	3 (25%)	8 (67%)
**Sugar free confectionery**	10	8 (80%)	2 (20%)	0 (0%)	0 (0%)	0 (0%)	10 (100%)	0 (0%)	0 (0%)	0 (0%)
**Cooking sauces**	10	3 (30%)	7 (70%)	10 (100%)	0 (0%)	10 (100%)	7 (70%)	3 (30%)	0 (0%)	0 (0%)
**Condiments**	8	7 (88%)	1 (12%)	0 (0%)	5 (63%)	5 (63%)	7 (88%)	1 (13%)	0 (0%)	0 (0%)
**Soup**	8	1 (13%)	7 (87%)	7 (88)	0 (0%)	7 (88%)	8 (100%)	0 (0%)	0 (0%)	0 (0%)
**Savory biscuits**	7	6 (86%)	1 (14%)	1 (14%)	3 (43%)	4 (57%)	4 (57%)	3 (43%)	0 (0%)	0 (0%)
**Fruit and nut**	5	2 (40%)	3 (60%)	3 (60%)	0 (0%)	3 (60%)	4 (80%)	0 (0%)	1 (20%)	0 (0%)
**Fish**	4	0 (0%)	4 (100%)	1 (25%)	0 (0%)	1 (25%)	4 (100%)	0 (0%)	0 (0%)	0 (0%)
**Stuffing**	2	0 (0%)	2 (100%)	2 (100%)	0 (0%)	2 (100%)	2 (100%)	0 (0%)	0 (0%)	0 (0%)
**Baked beans**	2	0 (0%)	2 (100%)	2 (100%)	0 (0%)	2 (100%)	2 (100%)	0 (0%)	0 (0%)	0 (0%)
**Total products (in out-of scope categories)**	435 (100%)	240 (55%)	195 (45%)	189 (43%)	39 (9%)	228 (52%)	355 (82%)	37 (9%)	30 (7%)	13 (3%)
**Total (% of all included products)**	676 (100%)	282 (42%)	398 (59%)	278 (41%)	79 (12%)	357 (53%)	575 (85%)	56 (8%)	32 (5%)	17 (3%)

Percentages (in brackets) are out of the total number of products in each category/row, unless stated.

1 Less healthy high fat sugar salt (HFSS) products were defined by a score of 4 or more using the UK Nutrient Profile Model [16].

2 Front-of-pack nutrition labels (FOPNL) types are categorized as either multiple traffic lights (MTLs) or others, which included non-MTL schemes such as monochrome or energy-only lozenge (as described in the UK Guidance) [5].

3 Inherent “red” traffic lights were calculated using product-level information and the UK multiple traffic light criteria [5].

4 Product categories in-scope of the regulations [14] (definitions in Supplemental Table 2).

5 Product categories considered out-of-scope of the regulations (for category definitions see Supplemental Table 3)

### Products in RAs: display of FOPNL and possession of iRTLs

**FIGURE 3:**
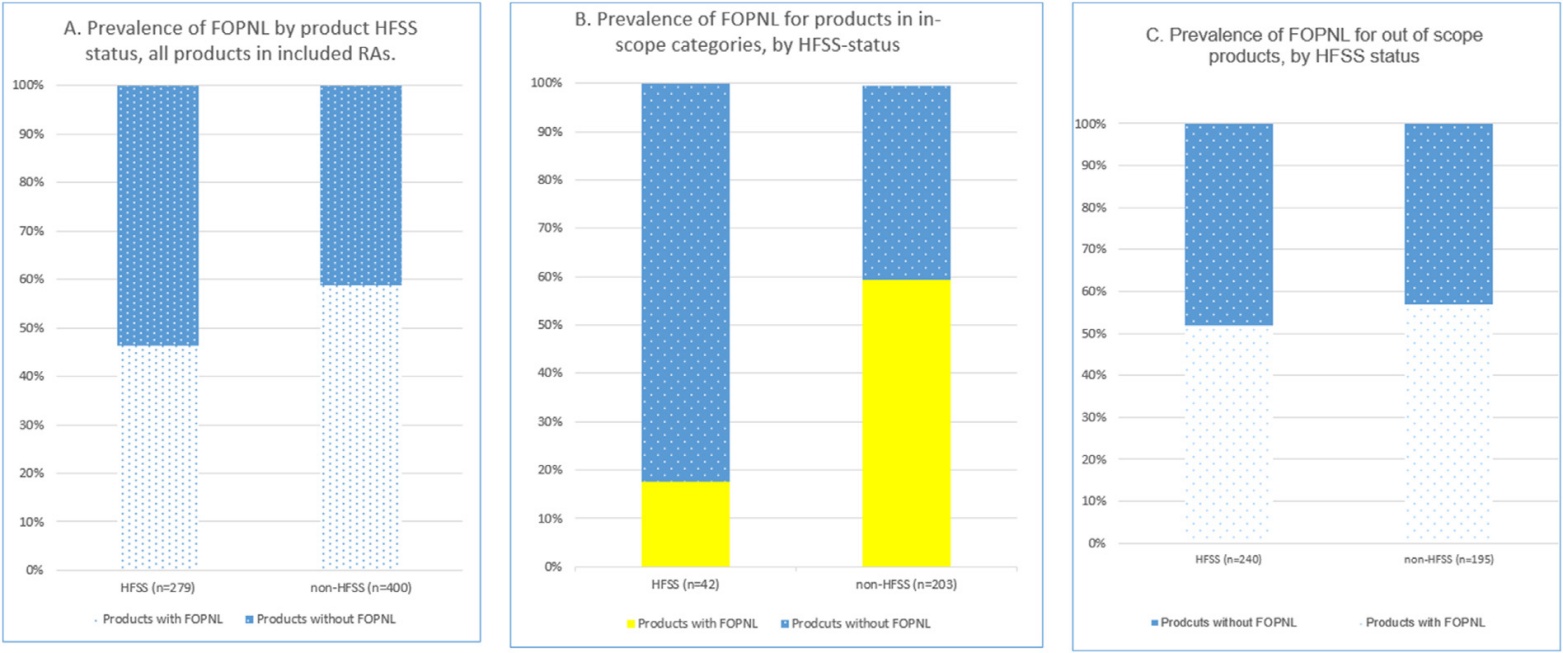
Prevalence of FOPNL on products in included restricted areas. (A) Prevalence of FOPNL across all products, categorized by HFSS status. (B) Prevalence of FOPNL on products in categories in-scope of the regulations, by HFSS status. (C) Prevalence of FOPNL on products in categories out-of-scope of the HFSS regulations, by HFSS status. FOPNL, front-of-pack nutrition labeling; HFSS, high fat, sugar, and salt.

**FIGURE 4:**
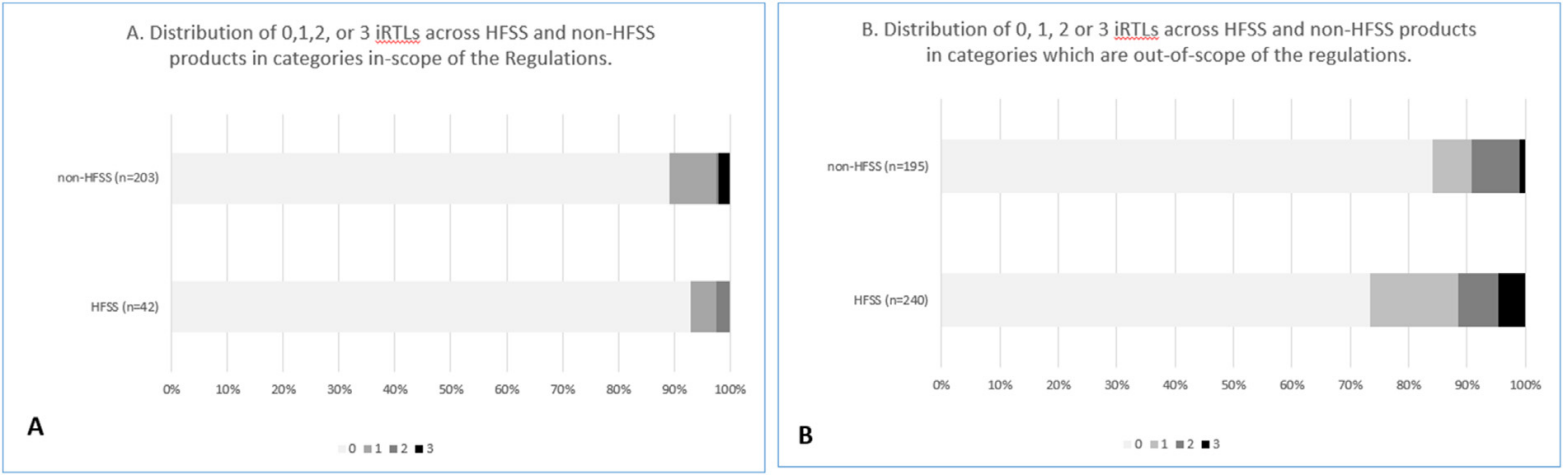
Prevalence of possession of inherent red traffic lights (iRTL), according to HFSS status of products in categories in-scope (A) and out-of-scope (B) of the regulations. HFSS, high fat, sugar, and salt.

Overall prevalence of FOPNL (that is, including MTLs, monochrome panels, etc.) across products in included RAs was 53%. FOPNL displayed as MTLs appeared on fewer products (41%) overall. Prevalence of FOPNL, including those displayed as MTL, also varied across product categories that were both in- and out-of-scope of the regulations, with FOPNL on 100% (all as MTLs) of both breakfast cereal and party food products sold in included RAs (Table 3). Overall, FOPNLs were found on a slightly higher proportion of all products that were non-HFSS (59%), compared with those that were HFSS (46%) but this was not statistically significant (X^2^ = 2.3, df = 1, *P* = 0.123) (Figure 3A). However, across products in those categories considered in-scope of the regulations, there was a significantly higher proportion of non-HFSS products displaying FOPNL (59%) compared with those classified as HFSS (18%) (X^2^ = 25.01, df = 1, *P* < 0.001) (Figure 3B). Furthermore, regardless of the display of FOPNL, 16% (*n =* 105) of products sold within the included in-store RAs possessed between 1 and 3 iRTLs, including both HFSS and non-HFSS products across categories that were both in (11%) and out (18%) of scope of the regulations (Figure 4A, Table 3). For example, across products that were out-of-scope of the regulations (that is, the majority of those in the included RAs), 28% of HFSS products and 17% of non-HFSS products possessed between 1 and 3 iRTLs (see Figure 4B).

## Discussion

### Summary of main findings, interpretation, and relation to the existing literature

This study of RAs in 3 supermarkets first outlines our step-wise approach to evaluating the in-store implementation of the new Food (Promotion and Placement) Regulations (England) 2021, at product-level, to provide the following findings. First, we show that the majority of the identified in-store RAs (54) displayed non-food products, and were not therefore included in the onward analysis. Among those RAs that were included, almost two-thirds (64%) of food products were from categories out-of-scope of (not covered by) the regulations, including pies and pastries, party food, etc. Together, both findings suggest that retail stores might take a partly category-based approach when implementing the new regulations, such that non-food and food products sold in RAs are likely to be from those outside the 13 in-scope categories. However, around a third (36%) of products sold in the included RAs were from those 13 in-scope categories (that is, breakfast cereals, cakes and cakes, confectionery, etc.) selected because these are known major contributors to children’s sugar and calorie intakes [14]. In comparison, a previous report conducted before the regulations were implemented found that 70% of products sold in various “prime” key in-store locations (that is, aisle and checkouts) were from such categories [19].

However, our study goes beyond product categories, and applies the UK NPM, as required by the regulations, to identify which products specifically are restricted (that is, according to HFSS status) within the included RAs. Prevalence of HFSS products that fell into those 13 in-scope categories was 17%. This suggests these stores were not in full compliance with the regulations, across the included RAs, at the time of our study. When compared with a previous (pre-regulation) in-store evaluation of products at checkouts (not aisle ends) that also used the UK NPM, 35% of products were classified as less healthy (HFSS) even within supermarkets with clear policies restricting less-healthy checkout foods [13].

It is also important to highlight that both HFSS and non-HFSS products were identified in the included RAs, including for categories in-scope of the regulations. For example, among the “sweet biscuits and bars” and “cakes and cupcakes” products included, all or most were deemed non-HFSS. The existence of these non-HFSS products in such categories may be due to industrial reformulation to alter nutrient and ingredient delivery, in line with those thresholds of the UK NPM, as described elsewhere [20]. Nonetheless, we also found that over half of the products in out-of-scope categories (that is, those not covered by the regulation) sold within the included RAs were HFSS (less healthy). Although this is likely due to the compositional nature of some categories of products (that is, fat and fat spreads), other out-of-scope categories including “pies, pasties, and Yorkshire puddings” and “party food” warrant highlighting. Products in these 2 categories alone made up 11% of those out-of-scope products in the included RAs, of which the majority (65%) were HFSS. Although our work did not collect data before the implementation of the regulations, it is possible that a consequence of this new policy is that more “out-of-scope” product categories are now being displayed in these key locations in-store.

The occurrence of out-of-scope products in RAs that are also “less healthy” is of further concern given that our findings also show that such permitted products may also not display FOPNL, without which consumers may be “in the dark” about which promoted products deliver “high” (red) levels of specific nutrients of public health concern. Indeed, we found that overall only around half of products sold in RAs display FOPNL, and not all of these were in the MTL format, as recommended by UK policy [4,5]. This prevalence of FOPNL specifically on products promoted in key in-store locations is a new finding we add to the current literature, and is similar to previous evaluations of the penetration (∼50% of products) of this voluntarily displayed product information in-store [21] and online [22,23]. However, in our study a further concern is that for products in in-scope categories, those classified as less healthy (HFSS) were significantly less likely to display FOPNL than non-HFSS products.

Finally, we show that there exists both overlap and misalignment between the 2 UK policies used here to evalauted product-level healthfulness, because some products categorized as “non-HFSS” were found to possess a number of iRTLs. This is likely due to differences between the criteria used in the UK MTL and UK NPM, also reported recently [22]. For example, for products in the regulated in-scope category “ready to cook meals or meal centers,” 73% displayed MTL and almost all products (96%) were non-HFSS, yet 20% of these products possessed ≥1 iRTL, and 9% possessed 3 iRTLs. Furthermore, this study now also suggests that many products in out-of-scope categories (that is, pies, pastries and Yorkshire puddings, party food, instant pasta, rice, noodles), which are allowed to be sold in RAs in key in-store locations regardless of HFSS status, can also possess a number of iRTLs indicating the delivery of “high” levels of fat, saturates, salt, or sugars. The simultaneous possession of iRTLs alongside the unreliable display of FOPNL, including MTLs across the permitted (out of scope) product categories surveyed here, has important implications for both consumers and industry. This is because the display of MTL FOPNL is known to encourage healthier choices, and drive industrial reformulation practices aimed at reducing the number of “red” traffic lights [24].

### Implications for research and future policy impact evaluation

To our knowledge this work is the first research evaluation of the in-store implementation of the new regulations and implies incomplete compliance with the regulations because some HFSS products were found to be present in in-scope categories in in-store RAs. Findings therefore imply the need for enforcement of the new regulation [25] and echo other research into the challenge of undertaking such enforcement given the required categorization, and complex product-level UK NPM calculations [20,26]. Our findings are also the first to highlight to policy makers the initial consequences of implementation of the new regulations, which mean it is now possible for RAs to legally display products that are HFSS yet in categories “out-of-scope” (that is, pies and pastries) and which may or may not display (voluntary) FOPNL indicating any “high” (red) levels of specific nutrients of public health concern. Conversely, policy makers should be aware that it is also now possible for some permitted non-HFSS products to also possess iRTLs, which again may or may not be (voluntarily) displayed on the front-of-pack.

Recommendations for policy makers now include the need for *1*) the regulations to go further and include additional in-scope categories, including pies and pastries, *2*) the United Kingdom’s existing voluntary FOPNL policy to become mandatory, to clearly show MTL FOPNL on products in RAs [27], and *3*) continued enforcement of the regulations, which the researchers suggest may be facilitated with new consumer-facing product labeling to indicate “HFSS status” or “NPM score” thereby aiding both enforcement activities and store-level implementation. Together with our 7-step approach, and detailed, extensive, in- and out-of-scope category descriptions, our findings now support future evaluation of the impact of the regulations [26] on consumers and industry, and future development of the policy that best supports consumer healthier food choices.

### Limitations and strengths of this study

This study is cross-sectional and as such findings do not reflect product changes in RAs at timepoints pre- to post-implementation of the new regulations. In addition, our product-level evaluation was based on included RAs in 3 stores in 1 UK city at a single time period throughout 2 major national holidays taking place in December and January, after the implementation of the regulations the previous October. As such, results may not reflect other (that is, non-seasonal) time frames in those supermarkets nor nationally in other stores, given that stores change their promotional displays frequently, and perhaps even daily. Nonetheless, we aimed to collect a sample of products sold in RAs, by including stores of varying sizes and those in different urban locations, reflecting 3 different major UK retailers. In addition, our manual approach to identification of RAs without using measuring tapes to record the exact distances specified in the regulations for certain types of RAs, and identifying products based on photographs and online records may be subject to human error. Although both may have led to the incorrect products or RAs being included, this is unlikely because the majority of included RAs were “aisle-end” types, identification of which is not reliant on checking distances. This approach, which is specifically related to the regulations and independent of industry involvement, will be of interest to enforcement authorities. However, we highlight that it was not the aim of this study to check the legal compliance of stores, but instead to capture and evaluate the nutritional and labeling nature of specific products in RAs in retail food environments.

Other limitations include the lack of (online) full nutritional and ingredient information for some products (that is, no information on fiber or percentage of fruit, vegetable, or nut ingredients), which hindered the application of the UK NPM. However, these labeling omissions are unlikely to sustainably change our classification of products as either “HFSS” or “non-HFSS” given that such fruit, vegetable, and nut content is required to be over 40% to “count” toward NPM score, reducing the risk of misclassifying the HFSS status of a product [21]. Finally, we placed individual products into categories (that is, either in- or out-of-scope), by using the regulation’s implementation guidance [14] together with published industry guidance on product-specific inclusions and exemptions [18] and product-level information from online supermarket records including description and ingredients. Although challenges still exist in categorizing some products, we believe this is the first research to report a full list of definitions of both in- and out-of-scope categories (see Supplemental Tables 2 and 3), which are now of use to enforcers, and also researchers seeking to reduce study heterogeneity in research evaluating the impact of the regulations on sales [7].

## Conclusion

There exists a varied prevalence of less-healthy (HFSS) products promoted in RAs within 3 stores across categories both in- and out-of-scope of the new regulations, shortly after their implementation in October 2022. Although a feature of healthier food retail environments, the display of voluntary FOPNL (that is, traffic lights) was found to be variable across HFSS/non-HFSS promoted products in categories both in- and out-of-scope of the regulations, including those that possessed but may not display “red” traffic lights indicating high levels of specific nutrients of public health concern. Although limited in the number of stores surveyed, this study is the first researcher-led evaluation of those recently regulated consumer-facing product promotions within key locations in-store food retail environments, with specific implications for future policy development and impact evaluation.

## Author contributions

The authors’ responsibilities were as follows – SGM, EH, LWW: designed the study; EH: conducted the research, analyzed data, and performed statistical analysis together with SGM who wrote the manuscript with contributions from EH and LWW; and all authors: read and approved the manuscript.

## Data availability

Data described in the manuscript will be made publicly and freely available by request to the corresponding author.

## Funding

The authors reported no funding received for this study.

## Conflict of interest

The authors report no conflicts of interest.
