# Supplementary material for: Prevalence of High Fat Sugar Salt Products, Labeling Characteristics, and Categories of Foods Sold within In-Store Restricted Areas: A Survey in 3 UK Supermarkets after the 2022 Implementation of the Food (Promotion and Placement) Regulations
Source: Curr Dev Nutr. 2024 Nov 20;9(1):104509. doi: 10.1016/j.cdnut.2024.104509 (PMC11681850; doi:10.1016/j.cdnut.2024.104509)
Supplement: Multimedia component 1 [file mmc1.docx]

Prevalence of high fat sugar salt (HFSS) products, labelling characteristics and categories of foods sold within in-store Restricted Areas: A survey in three UK supermarkets following the 2022 implementation of The Food (Promotion and Placement) Regulations.

**Hurst et al.**

**Supplementary Information**

**Table S1. Types and definitions of Restricted Areas (RAs) summarised from the Regulations (DHSC, 2023)**

**
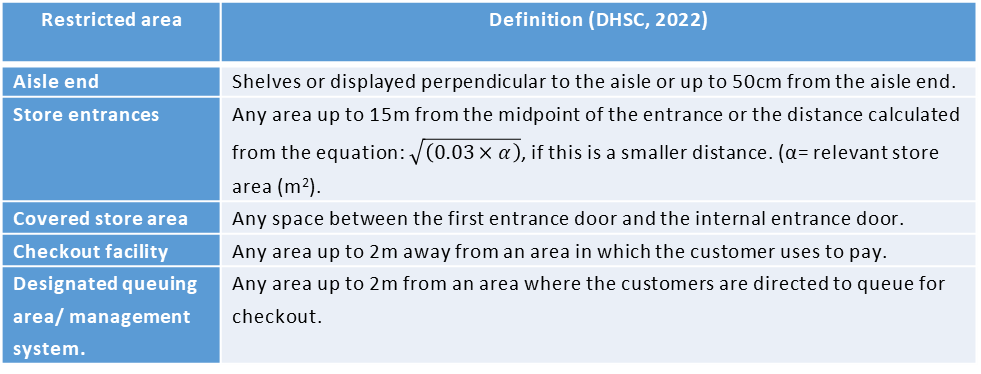
**

**Table S2: Descriptions and definitions of product categories in scope of the Regulation [14, 18].**

| **Product Category** | **Principal** | **Includes** | **Excludes** |
| --- | --- | --- | --- |
| **1 (Soft drinks)** | *“Prepared soft drinks containing added sugar ingredients”^1^* | Milk or milk alternative based drinks with added sugar or sweeteners  Fruit juice or smoothies with added sugar  Yogurt drinks with added sugar  Tea or coffee with pre-added sugar | Diet replacements *“alcoholic drinks and alcohol substitute drinks with more than 1.2% alcohol by volume”^1^* |
| **2 (Savoury snacks)** | “Savoury snacks whether intended to be consumed alone or as part of a complete meal”^1^, primarily, crisps or products eaten as a replacement to crisps. | *-“products made from potato, other vegetables, grain or pulses”^1^ and seaweed.*  *- “extruded, sheeted and pelleted products” ^1^*  *-“bagged savoury crackers, rice cakes or biscuits” ^1^*  -“Pork rind-based snacks” | Nuts of any form  Meat jerky |
| **3 (Breakfast cereals)** | Products that would be “*found in the ‘breakfast cereal’ aisle.”*^1^ | -“granola  -muesli  -ready-to-eat cereals  -porridge oats”^1^ | Cereal bars |
| **4 (Confectionary)** | Products that would be “*found in the ‘confectionery’ aisle*”^1^,included due to their ingredients that align with confectionary ingredients. | -Any form of chocolate  -Any sweets with added sugar  -Sweetened popcorn  -Chocolate coated nuts, seeds or fruit. | Sweet coated nuts |
| **5 (Ice cream and similar)** | “All dairy and non-dairy ice cream or ice cream alternatives”^1^ | -ice lollies  -sorbets  -frozen yoghurt  -water ices | Alcohol slushies |
| **6 (Cakes and Cupcakes)** | Any type of cake or similar | -Doughnuts  -Flapjacks  -Choux pastry desserts | -Cake decorations  -Cookies  -tarts |
| **7 (Sweet biscuits or bars)** | *“Sweet biscuits and bars based on one or more of nuts, seeds or cereal”^1^* | -cereal bars  -Sweet rice or corn cakes  -Ice cream cones |  |
| **8 (Morning goods)** | “Sweet pastries and buns, morning goods mixes and fruited bread”^1^ | Bagels  Brioche  Pancakes/waffles  Scones | “Savoury bread products”^1^ |
| **9 (Desserts and Puddings)** | All types of desserts | *“pies, tarts and flans, cheesecake, gateaux, dairy desserts, sponge puddings, rice pudding, crumbles, fruit fillings, powdered desserts, custards, jellies and meringues.”^1^* | -Plain meringue  -Dessert toppings and sauces |
| **10 (Sweetened yogurt)** | Yogurt sweetened in any form | -Probiotic yogurt  -Drinking yogurt with on liquid added. | Unsweetened yogurt |
| **11 (Pizza)** | Any pizza with topping | Flatbread pizzas | -Plain pizza bases  -Garlic bread |
| **12 (Fried potatoes)** | Potato products cooked in oil | *“Roast potatoes, potato and sweet potato chips, fries and wedges, potato waffles, novelty potato shapes (such as smiley faces), hash browns, rostis, crispy potato slices, potato croquettes.”^1^* | -Potatoes with butter  -Potato salads |
| **13 (Ready to cook meals or meal centres)** | *“Products that are marketed as ready for cooking or reheating without requiring further preparation and intended to be consumed as a complete meal” or “the main element of a meal”^1^*  Breaded or battered vegetables, fish or meat or substitute. | *-“Fresh pasta, rice or noodles with added ingredients and flavours, including filled or stuffed pasta”^1^*  -Ready meal without carbohydrate | -Cheese breaded or battered  -Any savoury pastry products (ie, pies or quiches)  -Meat, fish or alternatives cooked in a marinade or plain  -Party food |

^1^ Quotes in Italics from [14].

**Table S3: Descriptions and definitions of product categories out-of-scope of the Regulation [14, 18]**

| **Product category** | **Principal** | **Includes** | **Excludes** |
| --- | --- | --- | --- |
| **Nuts and Seeds** | Any nut or seed | “*Raw, coated, roasted or flavoured nuts and seeds (or mixes of these products).”^1^.* Also includes nut butters and purees. | Nuts or seeds coated in chocolate. |
| **Processed Meat/Meat** | Meat, either served plain, smoked or with a marinade, glaze, dressing, seasoning rub or similar accompaniment. | “‘*Meat’ includes meat and processed meat products such as ham, salami, chorizo, bacon, gammon, chicken thighs, grills, burgers (without a bun), sausages and steaks*”^1^. Also includes meat jerky. | -Breaded or Battered meat.  -Meat eaten as a ready meal or a meal centre (for example in a sauce but not marinade). |
| **Cheese and Cheese Dippers** | A product that is predominately cheese. | -Any cheese  *-“Breaded or battered cheese-based products intended to be consumed as a starter or side”^1^.*  -Cheese dippers are classed as cheese spread with a carbohydrate. |  |
| **Beverages** | Drinks without added sugar | - Drinks sweetened with only natural fruit or vegetable juice (including fruit juice)  - Milk, unsweetened milk-based drinks and unsweetened smoothies  *- “Alcoholic drinks and alcohol substitute drinks with more than 1.2% alcohol ABV by volume”^1^*  - Infant formula and follow up formula  - *“Total diet replacement as defined in foods intended for use in energy restricted diets for weight reduction”^2^.*  - Food for special medical purposes  - Tea or coffee without added sugar | - *“Cholesterol lowering drinking yoghurts”^2^*  - “Hot chocolate with added sugar / sweeteners”^2^  - “*Kombucha with added sugar (even when the sugar is used for fermentation purposes)”^2^*  - “*Sparkling juice drinks with added sugar*”^2^ |
| **Party Food** | Party food that is not intended to be consumed as a main meal | I.e mini or bite size version of savoury foods such as mini burgers and tempura prawns. |  |
| **Instant Pasta, Noodles and Rice** | Dried noodles, pasta and rice that require reconstitution prior to consumption |  |  |
| **Fruit** | Products predominately made of fruit. | -“*Dried fruit, fruit crisps or chips (includes both sweetened and unsweetened fruit ingredients).”^1^*  -Tinned or canned fruit | -Chocolate coated fruit  - Fruit fillings for desserts |
| **Plain Carbohydrates** | Carbohydrates with no included accompaniment or flavourings. | Pasta, bread and rice | -Instant rice, noodles or pasta.  -Fried potatoes |
| **Fat and fat spread** | Any form of fat | -Oils from plants or animals.  -Butter  -Margarine spread |  |
| **Vegetables/Pickles** | Vegetables without another element such as carbohydrate or protein element. | Includes vegetables preserved in:  -Pickle  -Oil | -Battered vegetables.  -Fried vegetables  -Vegetable juice |
| **Dessert Ingredients/Decoration** | Element of a dessert that does not make up the dessert on its own. | -Dessert toppings and sauces  -Icing  -Dessert and cake decorations. | Cake and other dessert mixes |
| **Spread and Sweet Spread** | Sweet spreads and yeast extract spreads. | -chocolate and biscuits spreads | -Fat only spreads  -Nut butters |
| **Gravy and Stock** | Gravy or stock with no other foods accompaniments. | -Pelleted gravy granules  -Stock cubes  -Fresh gravy and stock |  |
| **Pies, Pasties and Yorkshire Puddings** | Any savoury products which include pastry | -Pies, pasties, sausage rolls, tarts, tartlets, quiches, slices, lattices, plaits.  -Yorkshire puddings | Potato topped pies |
| **Sugar Free Confectionary** | Any sweets that do not have added sugar | Chewing gum, toffee sweets, hard boiled sweets.  Includes lozenges even with added sugar. |  |
| **Cooking Sauces** |  | -Tomato and other vegetable based sauces  -White sauces | Sauces combined with a carbohydrate, vegetable or protein. |
| **Condiments** | Sauce-like products added to food or eaten with food to add flavour | -Fruit sauces added to savoury foods such as cranberry or apple sauce  -Mustard  -Ketchup  -Mint sauce | -Cooking sauces  -sauces added to a sweet food |
| **Soup** | All soups | Fresh, canned, dried soup in a cup. |  |
| **Savoury Biscuits** | Savoury crackers not individually bagged as a replacement to crisps | -cream crackers  -water crackers  -oatcakes  -crackers for cheese  -crisp breads and breadsticks  -cheese twists  -large rice cakes | Bagged biscuits |
| **Fruit and Nut** | Products with only fruits and nuts | Fruit and nut trail mix without chocolate  Fruit and nut bars | Trail mixes with chocolate |
| **Fish** | Any form of fish or shellfish without any added carbohydrate, vegetable, or protein, | -Plain fish or shellfish  -Preserved fish, such as canned in brine or oil  -fish with flavouring | Battered or fried fish |
| **Stuffing** | A fresh or dry mixture used to stuff or accompany meat or vegetables. | Any stuffing products |  |
| **Baked Beans** | Baked beans cooked in tomato sauce | Tinned or fresh baked beans |  |

^1^ Quotes in italics from [14], ^2^ Quotes in italics from [18].
